# Suppressing non-radiative relaxation in a NIR single photon emitter: the impact of deuteration and temperature†

**DOI:** 10.1039/d4sc05517a

**Published:** 2024-11-27

**Authors:** Krishna Mishra, Zehua Wu, Christian Erker, Klaus Müllen, Thomas Basché

**Affiliations:** a Department of Chemistry, Johannes Gutenberg-University Mainz Mainz 55099 Germany thomas.basche@uni-mainz.de; b Max Planck-Institut für Polymerforschung Mainz 55099 Germany

## Abstract

The fluorescence quantum yield of organic NIR-emitters is typically limited by internal conversion (IC), restricting their applications in imaging and quantum technology. Here, we study the impact of deuteration and temperature on the emission properties of dibenzoterrylene (DBT) by bulk and single molecule spectroscopy. Based on simple photophysical modelling, we first clarify how IC affects the single molecule emission rate. Next, we show that deuteration of DBT leads to a concomitant increase in the fluorescence lifetime and quantum yield by up to 60%. This clear deuterium isotope effect indicates a significant contribution of C–H-vibrations in the IC process. The solvent-dependent changes in the IC rate of hydrogenated and deuterated compounds were found to follow the predictions of the energy gap law in the weak coupling limit. This view is supported by the very weak temperature dependence of the IC rate between 5 and 300 K. Our results not only shed light on the non-radiative relaxation of a topical polycyclic aromatic hydrocarbon, but also pave the way for single molecule quantum emitters with high emission yields in the NIR.

## Abstract 2

Deuteration suppresses the weakly temperature dependent internal conversion (IC) and turns dibenzoterrylene molecules into bright single photon emitters, in agreement with the predictions of the well-known energy-gap law.

## Graphical abstract

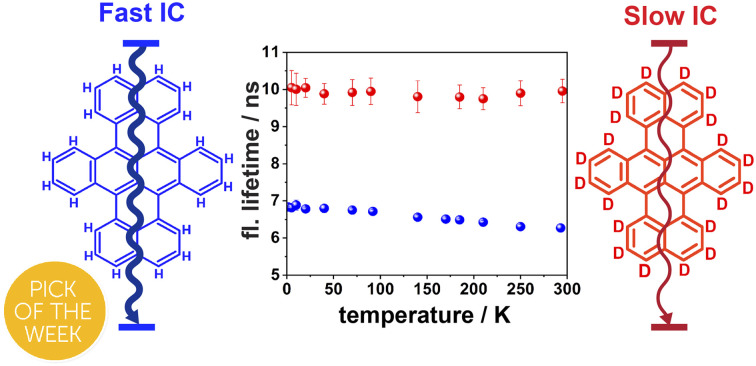

## Introduction

High fluorescence quantum yields of π-conjugated organic chromophores are desirable for their use in lighting,^1^ imaging^2^ and sensing^3^ or as non-classical single molecule light sources in the field of photonic quantum technologies.^4^ In general, when the emission wavelength of organic chromophores reaches the NIR region, the fluorescence quantum yield decreases due to internal conversion (IC) which increasingly competes with radiative decay.^5–8^ Typical quantum yields reported for different types of NIR dyes range between 0.2 and 0.3.^6–8^ The increase in the IC rate with decreasing energy of the S_1_–S_0_-transition is often thought to follow the energy-gap law (EGL).^9^ Recently, the implications and the two major aspects of the EGL as derived by Englman and Jortner^9^ have been scrutinized by Jang.^10^ Besides the assumption of weak coupling to molecular vibrations, the EGL model implies that the highest frequency vibrational modes serve as the main route for non-radiative decay. Moreover, it has been pointed out that within the framework of the EGL the IC rate should be largely independent of temperature.^10^ While experimental evidence for such behavior is lacking, very recent theoretical work employing a time-dependent formalism predicted that within the harmonic approximation, the IC rate for different organic dye molecules should be only weakly temperature dependent in the range of 0–300 K.^11^

The contribution of high frequency C–H-stretching vibrations in non-radiative decay processes such as IC or ISC (intersystem crossing) in polycyclic hydrocarbons (PAHs) has been a long-standing topic.^5,9,12^ In particular, this view is supported by the strong impact of deuteration on the T_1_ → S_0_ decay rate.^13^ The effect of deuteration on the IC rate of PAHs was reviewed recently by Ermolaev.^14^ Actually, the corresponding data basis is limited and mainly based on theoretical predictions or estimates from a few experimental data points.^12,15^ The main reason for this deficiency is the large S_1_–S_0_ energy gaps of the PAHs studied, which make it difficult to assess the effect of deuteration. More recent experimental and theoretical studies of different classes of NIR and SWIR (Shortwave Infrared) dyes have underscored the importance of high frequency C–H-vibrations.^16,17^ On the other hand a significant contribution from vibrational modes other than the highest frequency vibrational modes was reported for two flavylium dyes.^18^ These observations indicate the intricacies of the IC process and it remains elusive to what extent particular results can be generalized for different classes of organic dye molecules.

PAHs have been instrumental in the invention and development of single molecule spectroscopy (SMS).^19,20^ In light of their favorable properties, the study of quantum optical phenomena readily emerged as an important topic.^4^ Single photon emission from an individual PAH molecule was first revealed through photon antibunching measurements of pentacene in *p*-terphenyl at cryogenic temperatures.^21^ Later-on antibunching experiments were repeated with different PAHs under various conditions, leading to the concept of a molecular single photon source capable of emitting photons on demand.^22^ One important criterion of a single photon source is its brightness, which is directly related to the fluorescence quantum yield. Obviously, the ideal single photon emitter would emit exactly one photon after each excitation, requiring a quantum yield of unity. Yet, because the coupling of the electronic excitation to molecular vibrations mediates non-radiative relaxation pathways such as ISC and IC, typical quantum yields often are below unity. As mentioned before, this is especially true for fluorescence emission in the NIR region.

Because of its intense zero-phonon-line and very small ISC rate, the PAH dibenzoterrylene (DBT) has been used successfully in low temperature SMS.^23–25^ Recently, bulk experiments at room temperature revealed that the fluorescence quantum yield of DBT in different solvents is in the range of 10–35%,^26^ a typical value expected for a NIR-emitter.^6–8^ Moreover, it was shown at the bulk and single molecule level that with increasing solvent polarity the S_1_–S_0_ energy gap decreased, leading to a decrease in the fluorescence quantum yield and an increase in the IC rate, in accordance with the EGL.^26^ IC, being the dominating relaxation process, does not prevent single molecule detection, because electronically excited molecules are projected into the electronic ground state at a rate similar to that of the radiative transition. Recognizing the importance of the IC process, a crucial approach to improve the fluorescence quantum yield appears to decrease the IC rate without changing the other favorable photophysical properties of DBT.

**Scheme 1:**
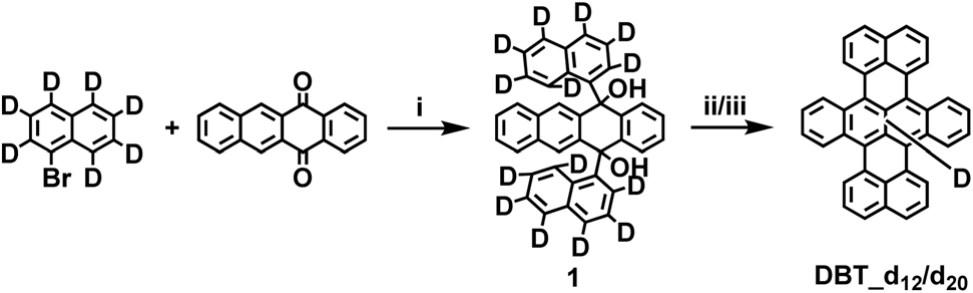
Synthetic route to DBT_d_12_ and DBT_d_20_. *Reagents and conditions*: (i) *n*-BuLi, 5,12-naphthacenequinone, THF, −78 °C, 60%; (ii) AlCl_3_, NaCl, 140 °C, 10 min, 20% for DBT_d_12_; (iii) AlCl_3_, chlorobenzene-d_5_, 100 °C, 1 h, 50% for DBT_d_20_.

Here, we study DBT at the bulk and single molecule level at temperatures between 5 K and 284 K. Using fully hydrogenated DBT (DBT_h_20_), we show that at very high excitation intensities, the photon emission rate becomes independent of the actual value of the IC rate. At typical excitation intensities below saturation, however, the emission rate strongly depends on the IC rate. As evidenced by the triplet population and decay rates, which are orders of magnitude smaller than the radiative and IC rates, the triplet bottleneck does play only a minor role in DBT. To boost the fluorescence quantum yield, we have deuterated DBT to different degrees (Scheme 1, and ESI†). With an increasing degree of deuteration, we find an increase in the fluorescence lifetime and quantum yield, which increase up to roughly 60% for fully deuterated DBT (DBT_d_20_) in toluene. Occasionally, for single molecules embedded in a Zeonex matrix, the quantum yield even reaches 75%. As had been shown previously for DBT_h_20_,^26^ DBT_d_20_ also follows the predictions of the energy gap law of internal conversion (EGL) at the bulk and single molecule levels when shifting the S_1_–S_0_ transition energy by different solvents/local environments. Temperature dependent measurements demonstrate that the fluorescence lifetime and the IC rate decrease only very weakly in the temperature range between 284 K and 5 K, indicating a minor improvement in the fluorescence quantum yield at low temperatures. Notably, the lack of temperature dependence of the IC rate which had not been shown before, seems to provide further evidence for the validity of the EGL.^9,10^ Altogether our results reveal a clear deuterium isotope effect, indicating a significant contribution of C–H(D)-vibrations in the IC process of a large PAH.

## Results and discussion

### Photophysics of DBT_h_20_

**Fig. 1:**
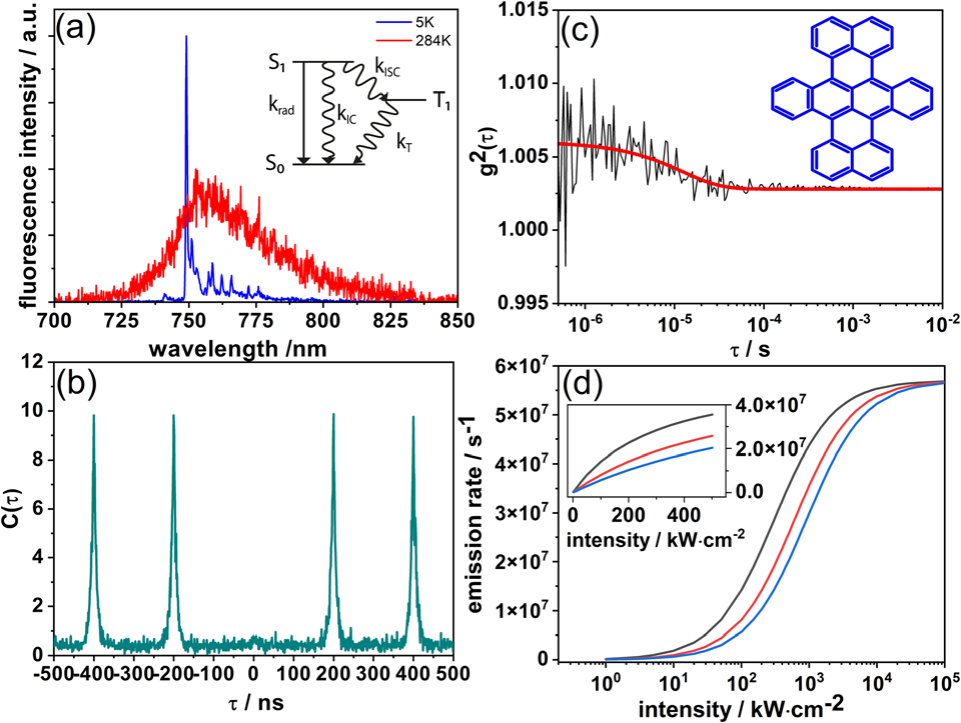
(a) Fluorescence emission spectra of the same single DBT_h_20_ molecule (in Zeonex) at 284 K (red) and 5 K (blue). The modulations in the red spectrum are due to NIR-etaloning from the CCD-chip. Inset: a reduced Jablonski diagram. (b) Coincidence count rate *C*(*τ*) after pulsed excitation of a single DBT_h_20_ molecule at room temperature. (c) Fluorescence intensity autocorrelation function *g*^2^(*τ*) of a single DBT_h_20_ molecule at room temperature. The red line represents a single exponential fit to the data. (Inset) Structure of DBT. (d) Simulation of the intensity dependence (eqn (1)) of the DBT_h_20_ fluorescence emission rate at different levels of the IC rate; black: *k*_IC_ = 0, red: *k*_IC_ = *k*_rad_, and blue: *k*_IC_ = 2 *k*_rad_.

In the initial experiments described here, we studied DBT_h_20_ at the single molecule level (measurement details, ESI†) to determine essential photophysical parameters. In Fig. 1(a) the fluorescence spectra of the same single DBT_h_20_ molecule at 284 K and 5 K, respectively, in a Zeonex matrix are shown. At low temperature (5 K) an intense [0,0]-zero-phonon line (ZPL) is observed together with a series of vibronic transitions. The vibronic features are consistent with recent reports in which DBT was investigated in crystalline and polycrystalline matrices.^24,27^

The coincidence count rate at room temperature for a single DBT_h_20_ molecule after pulsed excitation is presented in Fig. 1(b). As a typical signature of a single quantum emitter, the almost complete absence of the central peak at zero delay time signals high contrast photon-antibunching. We have also measured the fluorescence correlation function *g*^2^(*τ*) of single DBT_h_20_ molecules at long times under cw-excitation at room temperature (Fig. 1(c)). In each case, the contrast of the noisy correlation functions was very weak (Fig. 1(c)) as has been reported before at liquid helium temperature.^24^ Due to the weak contrast, long integration times of up to 30 minutes were needed for the correlation measurements, during which most of the molecules photo-bleached. Eventually, the correlation decay in the μs range could be analyzed for 15 DBT_h_20_ molecules in terms of the triplet kinetics, using standard expressions from the literature.^28–30^ A reduced Jablonski diagram with the various rates is given in the inset of Fig. 1(a). For the triplet decay rate, an average value of *k*_T_ = 5.5 ± 1.7 × 10^4^ s^−1^ was found which is in the same range as observed for single DBT molecules at low temperature.^24^ For the ISC rate and yield we determined *k*_ISC_ = 5.2 ± 3.5 × 10^3^ s^−1^ and *ϕ*_ISC_ = 3.1 ± 2.1 × 10^−5^, respectively. As seen from the larger errors, the ISC rate and yield varied appreciably from molecule to molecule while the triplet decay rate fluctuated much less. We assume that the large fluctuations of the ISC parameters are due to varying deviations from planarity for different DBT_h_20_ molecules which are known to affect ISC *via* σ–π coupling.^31,32^ The ISC yield appears to be at least one order of magnitude larger than that reported at low temperature, which is in line with similar observations for other PAHs such as terrylene^33^ and dibenzoovalene.^34^ In contrast, the triplet lifetime appears to be less sensitive to temperature. In the present context it is mainly relevant to consider the ISC rate, which even at room temperature, is orders of magnitude smaller than the radiative or IC rates (*vide infra*).

In contrast to ISC, in IC no bottleneck state is involved. Although straightforward, it is worthwhile to appreciate how IC affects a single molecule emission signal. With increasing excitation intensity and considering non-resonant excitation, the fluorescence emission rate *R*(*I*) saturates according to eqn (1),^28^1
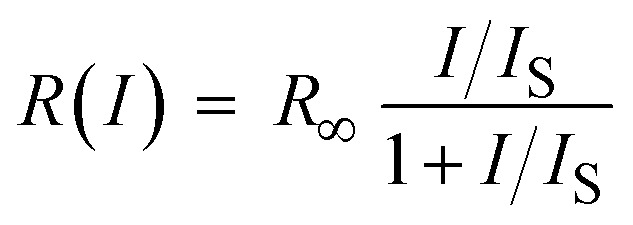
where *R*_∞_ is the fully saturated emission rate, *I* is the excitation intensity and *I*_S_ is the saturation intensity. Assuming *k*_rad_, *k*_IC_ ≫ *k*_ISC_, which is valid here, under non-resonant excitation, *R*_∞_ can be written as:^28^2
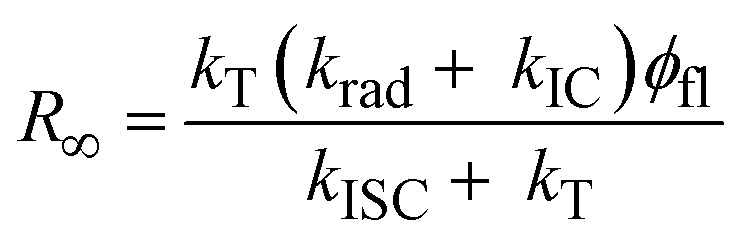
where *k*_rad_ is the radiative rate, *k*_IC_ is the IC rate, and *ϕ*_fl_ is the fluorescence quantum yield. Furthermore, the fluorescence decay rate *k*_fl_, which is the inverse of the fluorescence lifetime *τ*_fl_, is given by *k*_fl_ = *k*_rad_ + *k*_IC_. Inserting the average values of *k*_ISC_ and *k*_T_ into eqn (2) and using *k*_fl_ = 1.6 × 10^8^ s^−1^ and *ϕ*_fl_ = 0.39 obtained from single molecule measurements of DBT_h_20_ in Zeonex (Table 1), yields *R*_∞_ = 5.7 × 10^7^ s^−1^. The saturation intensity *I*_S_ is given by:^28^3
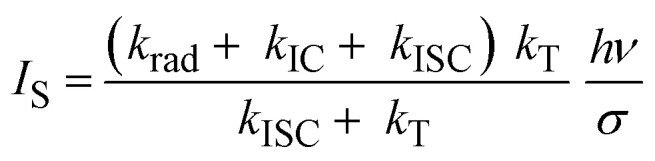
with *hν* being the photon energy and *σ* being the absorption cross section (computed from the extinction coefficient in solution) at an excitation wavelength of 670 nm. In Fig. 1(d) a calculation of *R*(*I*) as a function of the excitation intensity according to eqn (1) is presented for three values of *I*_s_, setting *k*_IC_ in eqn (3) to *k*_IC_ = 0, *k*_IC_ = *k*_rad_, or *k*_IC_ = 2 *k*_rad_. All other parameters were kept constant, with *R*_∞_ = 5.7 × 10^7^ s^−1^ (see above) and *k*_rad_ = 6.5 × 10^7^ s^−1^ (Table 1). As seen in Fig. 1(d), as the *k*_IC_ and *I*_S_ increase, a higher excitation intensity is required to reach a certain count rate. Irrespective of the IC rate, however, at very high excitation intensities, the emission rates converge towards *R*_∞_. Simplifying eqn (2) by using *k*_T_ > *k*_ISC_, it follows that under these conditions *R*_∞_ equals the radiative rate: *R*_∞_ = *k*_rad_ = *k*_fl_*ϕ*_fl_. This immediately reveals why *R*_∞_ remains constant for different values of *k*_IC_: a decrease in the quantum yield due to an increased IC rate is compensated by an increase in the total fluorescence decay rate. Nevertheless, we emphasize that unreasonably high excitation intensities would be necessary to approach *R*_∞_ when IC competes with radiative decay.

**Table 1:** Spectral and photophysical parameters of DBT_h_20_, DBT_d_12_, and DBT_d_20_ in various solvents and the Zeonex matrix^a^

Compound	Solvent	*λ*_em_/nm (cm^−1^)	*τ*_fl_/ns	*φ*_fl_	*k*_rad_/10^7^ s^−1^	*k*_IC_/10^8^ s^−1^
DBT_h_20_	Cyclohexane	743 ± 2 (13 459)	5.8 ± 0.1	0.38	6.5	1.1
	Toluene	780 ± 3 (12 820)	3.1 ± 0.1	0.16 ± 0.03	5.2	2.7
	DCM	830 ± 5 (12 048)	1.3 ± 0.1	0.05	3.8	7.3
	Zeonex	754 ± 9 (13 262)	5.9 ± 0.8	0.39	6.5	1
DBT_d_12_	Toluene	780 ± 2 (12 820)	4.6 ± 0.1	0.22	4.8	1.7
	Zeonex	750 ± 8 (13 333)	8.4 ± 1.4	0.51	6.1	0.6
DBT_d_20_	Cyclohexane	743 ± 2 (13 459)	8.2 ± 0.1	0.47	5.7	0.6
	Toluene	780 ± 3 (12 820)	5.5 ± 0.1	0.25 ± 0.05	4.6	1.4
	DCM	830 ± 5 (12 048)	2.3 ± 0.1	0.07	3	4
	Zeonex	749 ± 11(13 351)	10.2 ± 1.2	0.58	5.7	0.4

a *λ*_em_: emission maxima, *τ*_fl_: fluorescence lifetime, *φ*_fl_: fluorescence quantum yield (detailed calculation in ESI, Fig. S1), *k*_rad_: radiative rate, and *k*_IC_: internal conversion rate.

In Fig. S2† an experimental saturation curve is shown for a single DBT_h_20_ molecule. Using an estimated detection efficiency of 5%, an average value of *R*_∞_ = 3.1 ± 0.6 × 10^7^ s^−1^ is obtained from the saturation curves of 10 molecules, which is in reasonable agreement with the calculated value, considering the uncertainty in the detection efficiency. Since *k*_ISC_ is small and *k*_T_ > *k*_ISC_ holds, the triplet bottleneck has only a small impact on the fully saturated emission rate of DBT, which is close to that of an optical two-level system. Yet, this two-level system has an efficient non-radiative decay channel (IC), strongly affecting the emission rate at power levels used in typical experimental settings. In the following, we will illustrate the impact of deuteration and temperature on the fluorescence lifetime and quantum yield of DBT.

### Deuteration

To study the effect of deuteration, we prepared deuterated DBT through two different routes, both starting from deuterated bromonaphthalene as shown in Scheme 1. After synthesis of precursor 1, a cyclization reaction was conducted either in an AlCl_3_/NaCl melt or with AlCl_3_ in chlorobenzene_d_5_ as a solvent to obtain the deuterated DBTs. The efforts to obtain partially deuterated DBT_d_12_ and fully deuterated DBT_d_20_ yielded mixtures of compounds with different degrees of deuteration. As indicated by matrix-assisted laser desorption/ionization (MALDI) mass spectra, when an AlCl_3_/NaCl melt was utilized, the primary fractions consisted of DBT with 10–12 hydrogens substituted by deuterium. In contrast, the use of AlCl_3_/chlorobenzene-d_5_ predominantly resulted in DBT with 18–20 hydrogens replaced by deuterium. For the remainder, these mixtures will be called DBT_d_12_ and DBT_d_20_, respectively. The full details of the synthesis are provided in the ESI.†

**Fig. 2:**
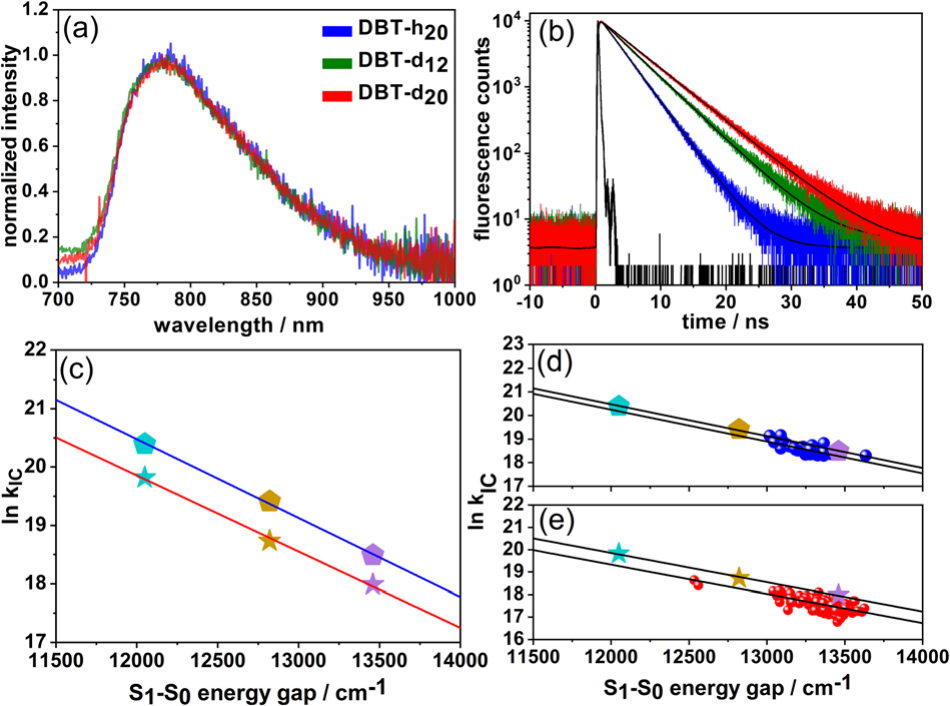
Bulk fluorescence spectra (a) and fluorescence decay curves (b) of DBT_h_20_ (blue), DBT_h_12_ (green) and DBT_d_20_ (red) in toluene. The black lines in (b) are single exponential fits to the data. (c) ln *k*_IC_ plotted against the S_1_–S_0_ energy gap (fluorescence maxima) for DBT_h_20_ (pentagons) and DBT_d_20_ (stars) in cyclohexane (cyan), toluene (maroon) and dichloromethane (magenta). The straight black lines are linear fits to the data. (d and e) Comparison of bulk solution and single molecule data. (d) The data of DBT_h_20_ from (c) are replotted (pentagons). The single molecule data of DBT_h_20_ in Zeonex appear as blue circles. (e) The data of DBT_d_20_ from (c) are replotted (stars). The single molecule data of DBT_d_20_ in Zeonex appear as red circles. The straight black lines are linear fits to the data.

The three compounds were dissolved in toluene and bulk absorption (Fig. S3†), fluorescence spectra and lifetimes were measured (Fig. 2(a) and (b)). While the absorption and fluorescence spectra did not change upon deuteration, the fluorescence lifetimes (quantum yields) did increase appreciably from 3.2 ns (0. 16) for DBT_h_20_ to 4.6 ns (0.22) for DBT_d_12_ to finally reach 5.5 ns (0.25) for DBT_d_20_ (Table 1). Within the experimental accuracy, for both quantities, an increase of ∼60% was obtained when moving from DBT_h_20_ to DBT_d_20_.

In the case of DBT_h_20,_ we observed a surprisingly strong fluorescence solvatochromism.^26^ With increasing solvent polarity, the S_1_–S_0_ energy gap was reduced, accompanied by a decrease in the fluorescence lifetime and quantum yield. The concomitant increase in the IC rate was found to be in full accordance with the predictions of the EGL.^26^ Based on this observation, we decided to compare spectral and photophysical parameters of DBT_h_20_ and DBT_d_20_ in different solvents.

In Table 1, the fluorescence lifetimes and quantum yields of DBT_h_20_ and DBT_d_20_ in cyclohexane, toluene and dichloro-methane (DCM) solutions are presented. From these quantities the radiative rates were obtained using *k*_rad_ = *ϕ*_fl_/*τ*_fl_. In addition, we calculated the radiative rates using the Strickler–Berg equation (Table S1†).^35^ For both compounds, these values are in reasonably good agreement with the values derived from the fluorescence quantum yield and lifetime.

With a decreasing S_1_–S_0_ energy gap, the radiative rates decrease for DBT_h_20_ and DBT_d_20_. One obvious contribution to the decrease in *k*_rad_ is the *ν*^3^ dependence of Einstein's spontaneous emission rate. Additional factors are difficult to assess but may be related to conformational flexibility or differences in transition dipole moments. Overall, the results indicate that the variations in the radiative rates do not depend significantly on H/D-substitution. To obtain the IC rates for DBT_h_20_ and DBT_d_20_ in the different solvents, we subtracted the radiative rates from the fluorescence decay rates *k*_fl_ = *τ*_fl_^−1^. As seen in Table 1, the IC rates in the deuterated compound are roughly a factor of 2 smaller in the different solvents. The increase in quantum yield, however, while being significant in each of the solvents, varies because of the interplay between the energy gap (solvent) dependence of the radiative and IC rate. In Fig. 2(c) ln *k*_IC_ is plotted against the S_1_–S_0_ energy gap (fluorescence maxima) of DBT_h_20_ and DBT_d_20_ in the three solvents. The energy gaps of the two compounds are almost identical in a given solvent and a linear dependence with almost identical slopes is observed, showing EGL behavior for DBT_h_20_ and for DBT_d_20_. This is also supported by the single-molecule measurements (Fig. 2(d) and (e)), which will be discussed later. Obviously, the main effect of deuteration is to increase the fluorescence lifetime and quantum yield of DBT by lowering the IC rate without significantly changing other photophysical or spectral parameters.

**Fig. 3:**
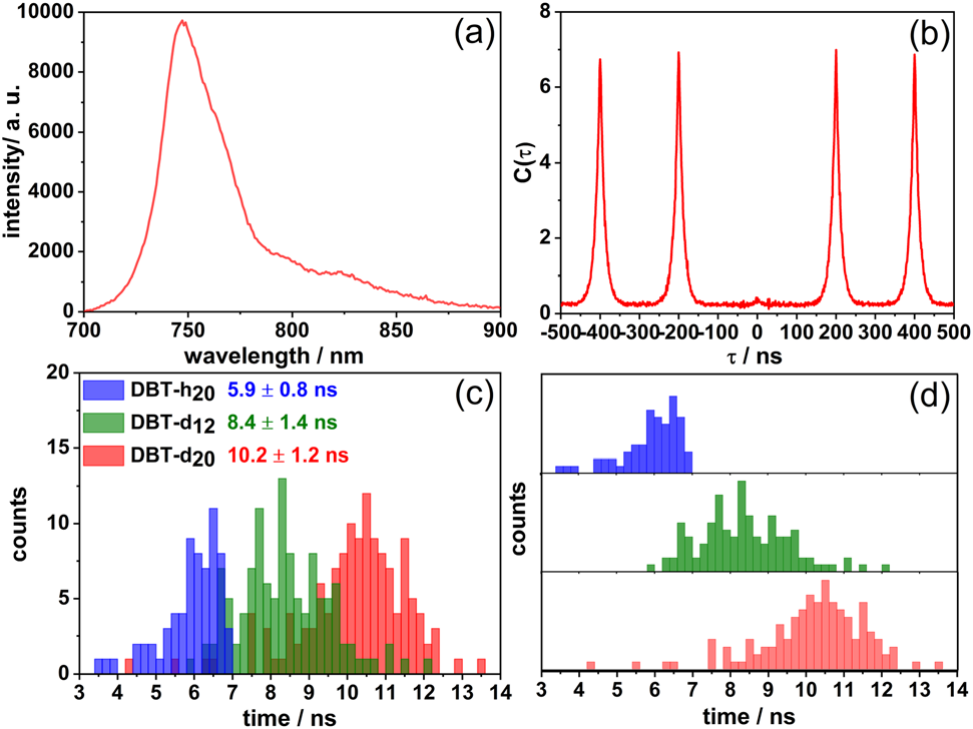
(a) Fluorescence spectrum of a single DBT_d_20_ molecule in Zeonex. (b) Coincidence count rate *C*(*τ*) after pulsed excitation of a single DBT_d_20_ molecule at room temperature. (c) Single molecule fluorescence lifetime distributions of DBT_h_20_ (blue), DBT_h_12_ (green) and DBT_d_20_ (red) in Zeonex. In (d) the distributions are plotted separately for clarity.

Single molecule studies of DBT_h_20_, DBT_d_12_ and DBT_d_20_ were performed in Zeonex films under an argon atmosphere at room temperature. In Fig. 3 a fluorescence spectrum (a) of a DBT_d_20_ molecule is shown along with the corresponding coincidence count rate (b) under pulsed excitation. For this molecule, the spectrum remained very stable before photobleaching, but in a number of cases appreciable spectral and intensity fluctuations were observed as reported before for DBT_h_20_.^26^ As was found for DBT_h_20_, the central peak was almost absent in the coincident count rate (Fig. 3(b)) demonstrating high-contrast photon antibunching. The latter result holds for the vast majority of the molecules studied. For all three compounds the distributions of emission maxima in Zeonex peak around 750 nm (Table 1). In Fig. 3(c) and (d) the distributions of the single molecule fluorescence lifetimes are displayed. The mean fluorescence lifetimes increase from 6.2 ns for DBT_h_20_ to 10.2 ns for DBT_d_20_, with the relative increase being ∼65%.

The fluorescence quantum yield could not be measured at the single molecule level. To provide an estimate for the quantum yield, we used the radiative rates obtained for DBT_h_20_ and DBT_d_20_ in the cyclohexane solutions, in which the emission maxima were closest to those in the Zeonex films. Since for DBT_d_12_ no value was determined, tentatively the average value of the other two compounds was used. The estimated quantum yields are given in Table 1. Inspecting the lifetime distribution of DBT_d_20_, occasionally molecules with fluorescence lifetimes in the range of 13 ns were found. In these cases, which most probably refer to molecules with full deuteration, the fluorescence rate (7.7 × 10^7^ s^−1^) approaches the radiative rate (5.7 × 10^7^ s^−1^), resulting in a quantum yield of ∼75% and turning DBT_d_20_ into an exceptionally bright single molecule NIR emitter.

In Table 1, the average single molecule IC rates of the three compounds in Zeonex are given which decrease by a factor of 2.5 from DBT_h_20_ to DBT_d_20_. For DBT_h_20_ and DBT_d_20_, we have plotted ln *k*_IC_ of single molecules as a function of their S_1_–S_0_ energy gaps (fluorescence maxima) in Fig. 2(d) and (e). A clear tendency towards EGL behavior is found. The larger scatter in the DBT_d_20_ data most probably reflects two (uncorrelated) contributions to the IC rate: on the one side the energy gap contribution and on the other side the degree of deuteration which can vary from molecule to molecule. In contrast, DBT_h_20_ is isotopically pure with respect to the hydrogen component. As seen in Fig. 2(d) and (e), the slopes of the linear fits are roughly the same as those for the bulk solution data. Yet, the ln *k*_IC_ and *k*_IC_ values, respectively, are slightly smaller in the solid Zeonex matrix as compared to the solution data. We speculate that in solution an additional small quenching contribution might operate.

### Temperature dependence

**Fig. 4:**
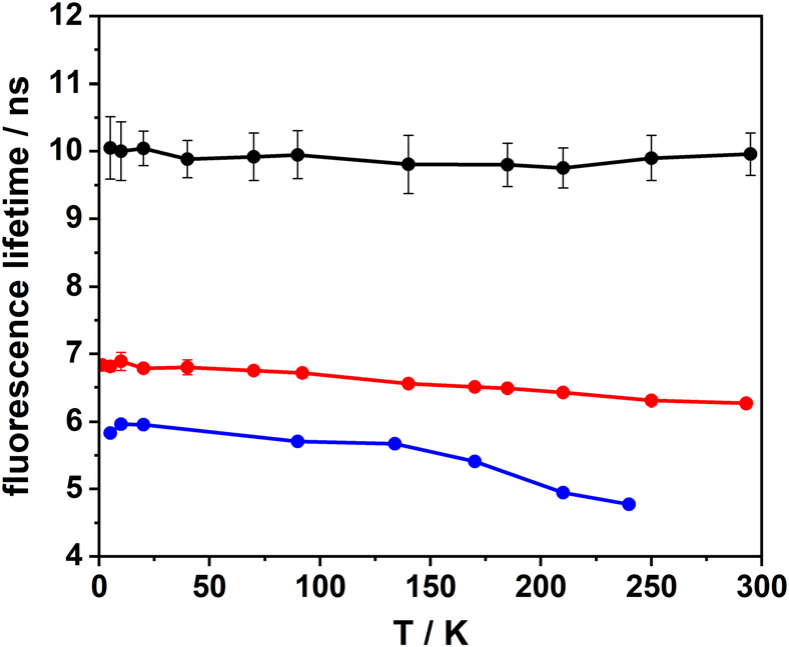
Temperature dependence of the fluorescence lifetime. Black circles: DBT_d_20_ in Zeonex, bulk sample; red circles: DBT_h_20_ in Zeonex, bulk sample; blue circles: DBT_h_20_ in Zeonex, single molecule. The drawn lines are guides to the eye only.

An intriguing question which to our knowledge has not yet been addressed concerns the temperature dependence of the IC rate in PAHs. In the context of single photon emitters, it will be crucial to know whether cryogenic temperatures lead to a decrease in the IC rate and a concomitant increase in the fluorescence quantum yield, as has been suggested recently.^36^ Along these lines, we have measured the fluorescence lifetimes of DBT_h_20_ and DBT_d_20_ in Zeonex as a function of temperature at the bulk and single molecule level. In the first step, these measurements were conducted with bulk samples, having DBT concentrations 2 to 3 orders of magnitude larger than those used for the single molecule samples. As seen in Fig. 4, the temperature dependence for DBT_h_20_ is weak leading to a small increase in the fluorescence lifetime from 6.3 ns at 284 K to 6.8 ns at 5 K. Below 100 K the lifetime remains almost constant. Within the error margins, there is no temperature dependence at all for DBT_d_20_. We note that in both cases the room temperature values of the fluorescence lifetime align well with the average values derived from the single molecule distributions (Table 1). Since the radiative rate should not depend on temperature, any changes in the fluorescence lifetime must be related to the IC rate. In the case of DBT_h_20,_ it decreases by roughly 10% from 1 × 10^8^ s^−1^ to 9 × 10^7^ s^−1^. Plotting the IC rate as a function of temperature (not shown) shows that it basically follows the fluorescence lifetime, remaining constant up to 100 K and growing weakly linear above 100 K. Considering DBT_d_20_, the IC rate (4 × 10^7^ s^−1^) is even smaller than the radiative rate (5.7 × 10^7^ s^−1^). Assuming that the IC rate would also decrease by only ∼10%, this would be difficult to resolve and most likely explains why no temperature dependence has been observed for DBT_d_20_.

In addition to the bulk measurements, fluorescence lifetimes (and spectra) were measured for a set of single DBT_h_20_ molecules in the temperature range between 5 K and 284 K. During temperature changes at elevated levels (*T* > 150 K), a large fraction of the molecules bleached irreversibly or could not be identified unambiguously due to thermal drift of the microscope. Moreover, appreciable fluctuations in the fluorescence lifetimes did occur, which were not related to the temperature but instead originated from spectral jumps and their consequences, as predicted by the EGL.^26^ A compelling example for such behavior is shown in Fig. S4.†

For some of the single molecules, the fluorescence lifetime did decrease with increasing temperature in a rather smooth fashion as exemplarily shown in Fig. 4. During warming-up, however, the emission of the molecules was often red-shifted, as seen in the case shown in Fig. 4 by about 8 nm in total with a particular increase above 100 K. Accordingly, the fluorescence lifetime did not decrease only because of the elevated temperature, but also because of the EGL. The red shifts may be related to a change in the coupling to the polymer host due to prolonged irradiation.

In recent work, the fluorescence quantum yield of single DBT_h_20_ molecules embedded in *p*-dichlorobenzene was measured at cryogenic temperatures.^36^ In this study values above 50% were reported for a large fraction of molecules, with some exceeding 70%. Considering the blue-shift of the emission in *p*-dichlorobenzene, a value of 50% does not seem to be too different from our estimated quantum yield of around 40% for DBT_h_20_ in Zeonex. On the other hand, quantum yields in the range of 70% and higher for a hydrogenated PAH emitting in the NIR region (>700 nm) appear to be quite unique and have not been reported at room temperature. To rationalize this finding, it was suggested that one possible reason may be a significant temperature dependence of the IC rate.^36^ Yet, the very weak temperature dependence of the fluorescence lifetime and IC rate, respectively, revealed in our study does not support such an explanation.

### Internal conversion and C–H(D)-vibrations

The results presented here show unambiguously that the replacement of hydrogen with deuterium has led to a substantial decrease in the IC rate underlining the importance of C–H(D)-vibrations in non-radiative relaxation from S_1_. Moreover, for DBT_h_20_ and DBT_d_20_ the EGL seems to hold at the bulk and single molecule level and for both compounds only a very weak temperature dependence of the IC rate was found. Recently, it has been reported that even in the presence of EGL behavior, vibrational modes other than the highest frequency modes (C–H-stretching modes) also make significant contributions to the IC rate.^10,18^ In light of these results, the observation of EGL behavior along with a significant deuterium isotope effect does not immediately exclude the contribution of low frequency modes for the case studied here.

Nevertheless, considering, for a moment, a contribution from C–H-stretching vibrations (ν̃ ∼ 3100 cm^−1^), roughly 4 quanta would match the energy gap of DBT in the various solvents. In the case of C–D-stretching vibrations (ν̃ ∼ 2200 cm^−1^), 6 quanta would be needed. Qualitatively, the larger the number of quanta needed to take up the excitation energy, the less probable the process will be, with a concomitant decrease in the IC rate. This view originates from the corresponding Franck–Condon factors which are small for low frequency vibrations and largest for high frequency vibrations.^5^ Thus, in accordance with other reports^16,17^ our results appear to indicate a contribution from high-frequency C–H(D)-stretching modes. Considering PAHs, we emphasize that our findings are one of the very few examples where the often-postulated participation of such vibrations align with the experimental results.

## Conclusions

Deuteration was shown to turn single DBT molecules into bright single photon emitters with emission yields reaching up to 75%. In general, this is an exceptionally large value for an organic dye molecule emitting in the NIR region at 750 nm. More common values for NIR emitters are around 20%, as exemplified by the reference compounds used in this study with reported quantum yields of 10% (ATTO 740) or 28% (HITCI) (Fig. S1†). Moreover, taking the longest fluorescence lifetimes observed for single DBT_d_20_ molecules (13 ns), the low temperature zero-phonon line would become as narrow as 12 MHz converting DBT into a high-quality oscillator with promising properties towards quantum optical applications.^4^ Although the contrasts of photon antibunching appeared to be quite similar for DBT_h_20_ and DBT_d_20_, we note that photons can be more efficiently extracted from a system where the IC rate – compared to a roughly constant radiative rate – is smaller.

Besides improving the properties of a single photon emitter, our results shed light on fundamental aspects related to the IC process and the applicability of the EGL. The energy gap dependence of the IC rate as well as the lack of temperature dependence – shown here for the first time – suggest that the EGL in its original form^9^ adequately describes the experimental data from DBT_h_20_ to DBT_d_20_. This conformity, together with the significant deuterium isotope effect, implies that high frequency C–H(D)-stretching modes should be a major player in non-radiative relaxation *via* IC. At present, however, a contribution from low frequency modes cannot be excluded solely based on our results. Given the ever-increasing capabilities in the quantum-chemical description of the IC process of large molecules, we hope that our comprehensive study will also stimulate an advanced theoretical treatment of the system and help clarify which vibrational modes are involved. The corresponding insights might also allow for clarifying whether a theoretical description of the IC process can be generalized for a larger class of compounds.

## Data availability

All data supporting this study are available in the article and ESI.†

## Author contributions

Conceptualization: TB; synthesis: ZW and KMü; data curation: KM and CE; analysis: KM, CE, and TB; investigation: KM and CE; supervision: TB and KMü; validation: all authors; visualization: KM and CE; writing original draft: TB, KM, ZW, and CE; review & editing: all authors; funding acquisition: TB.

## Conflicts of interest

There are no conflicts to declare.

## Supplementary Material

SC-OLF-D4SC05517A-s001
