# Supplementary material for: Suppressing non-radiative relaxation in a NIR single photon emitter: the impact of deuteration and temperature
Source: Chem Sci. 2024 Nov 27;16(1):90–7. doi: 10.1039/d4sc05517a (PMC11600331; doi:10.1039/d4sc05517a)
Supplement: SC-016-D4SC05517A-s001 [file SC-016-D4SC05517A-s001.pdf]

Supplementary Information (SI) for Chemical Science.
This journal is © The Royal Society of Chemistry 2024

Supporting Information

Suppressing non-radiative relaxation in a NIR single photon emitter: The impact of deuteration and temperature.

Krishna Mishra[a], Zehua Wu[a], Christian Erker[a], Klaus Müllen[a,b], Thomas Basché[a]*

[a]Department of Chemistry, Johannes Gutenberg-University, 55099 Mainz, Germany

[b]Max Planck-Institut für Polymerforschung, 55099 Mainz, Germany

E-mail: thomas.basche@uni-mainz.de

Contents	Page No.
Materials and synthesis details	S2
Instrument and measurement details	S3
Determination of the fluorescence quantum yield	S5
Single molecule emission rate as a function of excitation intensity	S6
Absorption spectra of DBT_h_{20}, DBT_d_{12}, DBT_d_{20} in toluene	S6
Calculation of radiative rates by the Strickler-Berg equation	S7
Single molecule spectral and lifetime fluctuations	S8
References	S9

1. Materials and synthesis details

All chemicals and solvents were purchased from Aldrich, Acros, Fisher, or TCI and used as received without further purification unless otherwise noted. Mass spectra were recorded by atmospheric pressure chemical ionization (APCI) mass spectrometry on a MicrOTOF-QII instrument and by matrix-assisted laser decomposition/ionization time-of-flight mass spectrometry (MALDI-TOF MS) using 7,7,8,8-tetracyanoquinodimethane (TCNQ) as matrix on a Bruker Reflex II-TOF spectrometer.

Synthetic route to DBT_d_{12} and DBT_d_{20}

Deuterated **DBTs (DBT_d_{12}** and **DBT_d_{20})** were synthesized by two routes as shown in scheme 1 in the main text. The precursor **1** was prepared by adding 5,12-naphthacenequinone to lithiated 1-bromonaphthalene-d_7 at -78°C. In the following cyclization step, **1** was treated with either a $AlCl_3$/NaCl melt or $AlCl_3$ in chlorobenzene-d_5. As indicated by mass spectra, rather than a single product, both methods yielded compounds with different degrees of deuteration ranging from m/z = 481.1848 to 496.2840. This can be attributed to H/D exchange between the target compounds and/or the solvent during the cyclization reaction in the presence of excessive Lewis acid $AlCl_3$. The mixture (**DBT_d_{12}**) obtained from the $AlCl_3$/NaCl melt was characterized by a wide molecular weight distribution from **DBT_d_5 to** completely deuterated **DBT_d_{20}**, the main products **d_{10}**, **d_{11}** and **d_{12}** making up a total of ~ 42 %. The mixture (**DBT_d_{20}**) obtained with chlorobenzene-d_5 as solvent gave rise to a narrower molecular weight distribution ranging from **DBT_d_{12}** to **DBT_d_{20},** the main products **d_{18}**, **d_{19}** and **d_{20}** making up ~ 62 %.

5,12-Bis(naphthalen-1-yl-d7)-5,12-dihydrotetracene-5,12-diol (1)

Under N_2 atmosphere, n-BuLi (1 mmol in n-hexane) was added dropwise to a solution of 1-bromonaphthalene-2,3,4,5,6,7,8-d_7 (213 mg, 1 mmol) in dry tetrahydrofuran at -78 °C. After stirring at -78 °C for 1 hour, 5,12-naphthacenequinone (85 mg, 0.3mmol) was added to the solution. The temperature was slowly raised to room temperature, and the reaction mixture was stirred overnight. The reaction was quenched by adding distilled water (200 mL), and the mixture was extracted with ethyl acetate (3×100 mL). By removing solvent under reduced pressure, the crude product of **1** was obtained (308 mg, 60%) and used in the next step without purification. HRMS (APPI+) *m/z*: calcd for C38H12D14O2 [M+]: 528.281; Found: 528.282.

7,8:15,16-dibenzoterrylene_d_{12} (DBT_d_{12})

Under N_2 atmosphere, compound **1** (52.8mg, 0.1mmol) was introduced into a melt of 0.5 g of sodium chloride and 2 g of aluminum chloride at 140°C. After 10 min of stirring, the mixture was cooled down and quenched with deuterium oxide (10 mL). The precipitate was collected by filtration. A deep green product was obtained (9.8 mg, 20%) after purification by preparative GPC column with THF as eluent. HRMS MALDI-TOF (TCNQ): calculated for $C_{38}D_5H_{15}$ (d5) m/z = 481.1879, $C_{38}D_6H_{14}$ (d6) m/z = 482.1942, $C_{38}D_7H_{13}$ (d7) m/z = 483.2004, $C_{38}D_8H_{12}$ (d8) m/z = 484.2067, $C_{38}D_9H_{11}$ (d9) m/z = 485.2130, $C_{38}D_{10}H_{10}$ (d10) m/z = 486.2193, $C_{38}D_{11}H_9$ (d11) m/z = 487.2255, $C_{38}D_{12}H_8$ (d12) m/z = 488.2318, $C_{38}D_{13}H_7$ (d13) m/z = 489.2381, $C_{38}D_{14}H_6$ (d14) m/z = 490.2444, $C_{38}D_{15}H_5$ (d15) m/z = 491.2507, $C_{38}D_{16}H_4$ (d16) m/z = 492.2569, $C_{38}D_{17}H_3$

(d17) m/z = 493.2632, $C_{38}D_{18}H_2$ (d18) m/z = 494.2695, $C_{38}D_{19}H_1$ (d19) m/z = 495.2758, $C_{38}D_{20}$ (d20) m/z = 496.2820; found m/z = 481.1848, 482.1933, 483.1997, 484.2066, 485.2127, 486.2191, 487.2255, 488.2313, 489.2378, 490.2440, 491.2500, 492.2557, 493.2620, 494.2686, 495.2757, 496.2840. MP: 308.5-309.5 ℃.

7,8:15,16-dibenzoterrylene_d$_{20}$ (DBT_d$_{20}$)

Under N_2 atmosphere, compound **1** (52.8mg, 0.1mmol) and aluminum chloride (0.4g, 3.1 mmol) were mixed in chlorobenzene-d5 (5 mL) and stirred at 100 °C for 1h. After cooling down, the reaction was quenched with deuterium oxide (10 mL). The solvent was evaporated under vacuum. A deep green crude product was obtained (24.5 mg, 50%) after purification by preparative GPC column with THF as eluent. HRMS MALDI-TOF (TCNQ): calculated for $C_{38}D_{12}H_8$ (d12) m/z = 488.2318, $C_{38}D_{13}H_7$ (d13) m/z = 489.2381, $C_{38}D_{14}H_6$ (d14) m/z = 490.2444, $C_{38}D_{15}H_5$ (d15) m/z = 491.2507, $C_{38}D_{16}H_4$ (d16) m/z = 492.2569, $C_{38}D_{17}H_3$ (d17) m/z = 493.2632, $C_{38}D_{18}H_2$ (d18) m/z = 494.2695, $C_{38}D_{19}H_1$ (d19) m/z = 495.2758, $C_{38}D_{20}$ (d20) m/z = 496.2820; found m/z = 488.2329, 489.2361, 490.2457, 491.2510, 492.2574, 493.2641, 494.2679, 495.2773, 496.2824. MP: 308.5-309.5 ℃.

DBT_h$_{20}$ was synthesized and purified according to prescriptions from the literature.[1]

2. Instrument and measurements details

Bulk absorption, fluorescence, and lifetime measurements

Absorption spectra were recorded on two different UV-VIS spectrophotometers (Lambda850, PerkinElmer; Duetta, Horiba). Corrected emission spectra were taken with a spectrofluorometer (Duetta, Horiba) with sensitivity up to 1100 nm. Fluorescence decays (TCSPC: time correlated single photon counting) were acquired on a spectrofluorometer (Fluorolog-3, Jobin Yvon) modified to our needs with a pulsed fiber laser (YSL SC-OEM + YSL VLF) and a hybrid detector (PicoQuant PMA Hybrid 50), both connected to a TCSPC module (PicoQuant PicoHarp 300). The temporal resolution of the set-up was ~ 0.3 ns.

Single molecule measurements

Thin-film samples for the single molecule experiments were prepared by spin-coating toluene/Zeonex-330R solutions with DBT concentrations from 10^{-9} M to 10^{-10} M onto carefully cleaned glass cover slides. For the temperature-dependent “bulk” measurements, concentrations in the range of 10^{-6} M to 10^{-7} M were used.
The room temperature experiments were conducted on a home-built confocal fluorescence microscope. The excitation source was a pulsed white light fiber laser (5 MHz, SC-OEM, YSL Photonics, China) followed by a variable optical filter (YSL VLF) set to ~670 nm in case of pulsed excitation, or a 685 nm solid state laser (Toptica iBeam-smart 685-S) in case of cw excitation. After passing a single-mode fiber, the respective laser light was recollimated and then focused onto the polymer films using a microscope objective (Plan-Apochromat 63x, NA = 1.4, oil immersion, Zeiss, Germany). The samples were flushed with argon to prevent premature photobleaching of the DBT molecules. The excitation power measured in front of the objective was, except for the saturation measurements, always set to 50 µW. The fluorescence emission

was collected by the same objective and separated from the excitation light by a 80:20 beamsplitter or a 699 nm dichroic beamsplitter and a 700 nm long-pass filter. After passing a 50:50 beamsplitter, the light was either focused onto the entrance slit of a spectrograph (Acton SpectraPro 300i) equipped with an EM-CCD camera (Andor iXon 888 or Princeton ProEM-1024BX3) or through an additional 50:50 beamsplitter onto 2 APDs mounted in a Hanbury-Brown-Twiss configuration. Single molecule emission spectra were recorded with either a 50 lines/mm or a 150 lines/mm grating.

Temperature dependent measurements down to 5 K were conducted with a separate home-built low-temperature confocal fluorescence microscope. The excitation source was the same pulsed laser system as mentioned above. The collimated light-beam was expanded to ~8mm and then routed through a telecentric scanning-mirror system into a cryostat (Janis Research Company SVT-10CNDT). After passing the cryostat windows, the excitation light was focused onto the sample using a microscope objective (Melles Griot, 01F/OAS017, 60x, NA = 0.85). The fluorescence light was separated using an 80:20 beamsplitter, passed through a pinhole, filtered by a 700nm long-pass filter and then divided onto 2 APDs and the entrance slit of a spectrograph (Andor Shamrock SR750) equipped with a CCD camera (Andor Newton EM-CCD), equivalent to the method used in the room temperature measurements. A 150 lines/mm grating was used in this case.

In both setups the APD signals were fed into a PicoHarp 300 or HydraHarp 400 module (PicoQuant), which records the absolute and relative arrival time (relative to the excitation pulse) of the individual photons. This enabled the subsequent calculation of fluorescence intensity time traces as well as fluorescence decay curves and the coincidence count rate under pulsed excitation. The time resolution of the set-ups was ~ 0.6 ns. Under cw-excitation the fluorescence intensity autocorrelation function at long times was calculated from the photon arrival times.

Temperature control from 5-293K was achieved by establishing a helium gas flow, which was first adjusted in a way that the sample was at a few Kelvins below the desired temperature. Then the heater of the cryostat, which was controlled by an electronic temperature controller (LakeShore 330), was activated and the helium flow fine-tuned, to stabilize the heater power roughly in the middle of its range. After each setting of a new temperature, the setup was allowed 30-60min to reach a stable equilibrium before any measurements were done.

3. Determination of the fluorescence quantum yield

Following standard protocols, and as described before [2], the fluorescence quantum yields of DBT_h_{20} and DBT_d_{20} in toluene were measured against two reference compounds (HITCI, ATTO740) with known quantum yields (HITCI: 28 % [3] ATTO740: 10 % [4]). The reference compounds were selected to have good spectral overlap with the emission spectrum of DBT (Figure S1 (a)). As commonly observed [5] and verified by measurements of solutions bubbled rigorously with argon, neither the fluorescence quantum yields, nor the fluorescence lifetimes did change in the presence of oxygen. To minimize the effects of reabsorption, we performed concentration-dependent measurements of the fluorescence intensity of the DBT and the reference compounds. Fluorescence intensity was plotted against the absorbance and a corresponding data set for DBT_h_{20} is exemplarily shown in Figure S1 (b).

The fluorescence quantum yield of the DBTs was calculated according to equation S1:

$$\phi^{DBT} = \phi^{ref} \frac{m^{DBT}}{m^{ref}} \left(\frac{n^{DBT}}{n^{ref}}\right)^2 \quad \text{(S1)}$$

ϕ^i are the quantum yields of the DBT and reference compounds, m^i the slopes of the linear fits to the data points in Figure S1 (b), and n^i the refractive indices of the solvents. In accordance with former measurements [2], we determined fluorescence quantum yields of 12 % and 20 % for $\phi^{\mathrm{DBT\_h20}}$ against HITCI and ATTO740, respectively. Similarly, In the case of $\phi^{\mathrm{DBT\_d20}}$ 20 % and 31 % were obtained. Since both values are not too different and there is no obvious criterion for which of the two values is more reliable, the average values have been taken for further discussion.

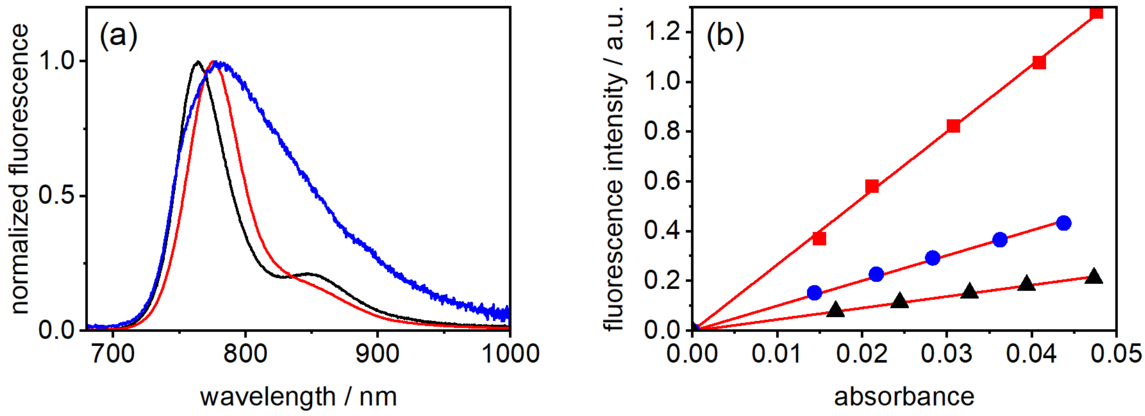

Figure S1. (a) Normalized emission spectra for DBT_h20 in toluene (blue, c = 3·10^{-6} mol/l), HITCI in ethanol (red, c = 8·10^{-7} mol/l) and ATTO740 in PBS buffer (black, pH: 7.4; c = 1·10^{-6} mol/l). In each case, the excitation wavelength was set to 670 nm. (b) Integrated fluorescence intensity versus absorbance (at 670 nm) for DBT_h20 in toluene (blue dots), HITCI in ethanol (red squares) and ATTO740 in PBS buffer (black triangles).

4. Single molecule emission rate as a function of excitation intensity

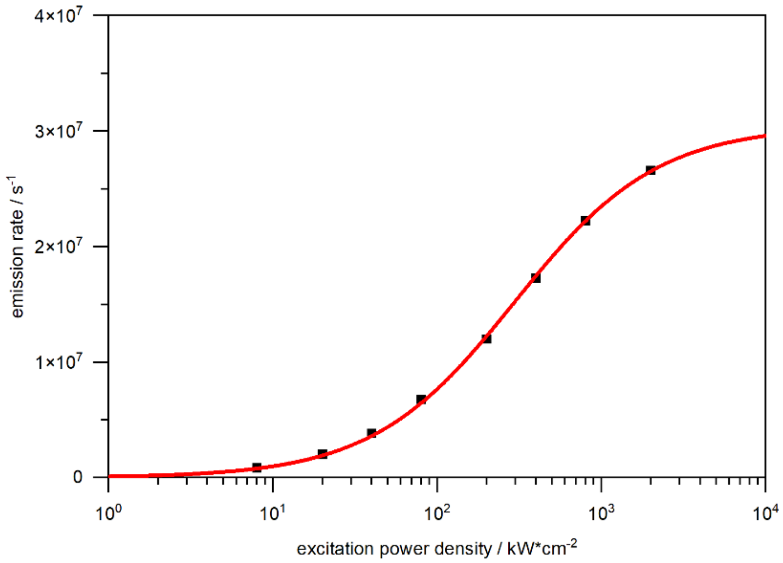

Figure S2: Fluorescence emission rate of a single DBT_h_{20} molecule at room temperature as a function of the excitation intensity. The red line is a fit of Equation 1 to the data.

5. Absorption spectra of DBT_h_{20}, DBT_d_{12}, DBT_d_{20}

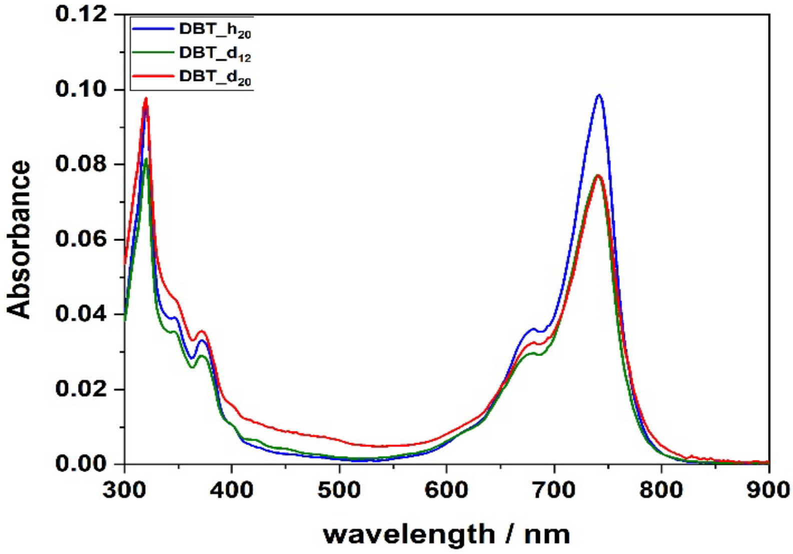

Figure S3. Absorption spectra of DBT_h_{20} (blue), DBT_d_{12} (green), and DBT_d_{20} (red) in toluene. Concentrations were in the range of ~2-3*10^{-6} M.

6. Calculation of radiative rates by the Strickler-Berg equation

$$k_{rad}^{cal} = \frac{1}{\tau_{rad}} = 2.88 \cdot 10^{-9} \cdot n^2 \cdot \langle \tilde{\nu}_f^{-3} \rangle_{av}^{-1} \cdot \int \frac{\varepsilon(\tilde{\nu})}{\tilde{\nu}} d\tilde{\nu} \quad \text{(S2)}$$

The radiative rates k_{rad}^{cal} have been calculated by the Strickler-Berg equation (S2). $\varepsilon(\tilde{\nu})$ is the extinction coefficient, $\tilde{\nu}$ the frequency in wavenumbers, $I(\tilde{\nu})$ the intensity of the fluorescence spectrum and n the refractive index of the solvent. $\langle \tilde{\nu}_f^{-3} \rangle_{av}^{-1}$ is the reciprocal of the mean value of $\tilde{\nu}^{-3}$ in the fluorescence spectrum. By integrating the absorption and emission spectra of DBT in the various solvents, the values of k_{rad}^{cal} provided in Table S1 have been obtained. They are in reasonably good agreement with the "experimental" values as given in table S1 which were derived from the fluorescence quantum yield and lifetime.

Table S1

Compound	Solvent	k_{rad}^{cal} /10^7 s^{-1}
**DBT_h$_{20}$**	Cyclohexane	5.4
	Toluene	5.8
	DCM	3.5
**DBT_d$_{12}$**	Toluene	5.2
**DBT_d$_{20}$**	Cyclohexane	4.3
	Toluene	4
	DCM	3.1

7. Single molecule spectral and lifetime fluctuations

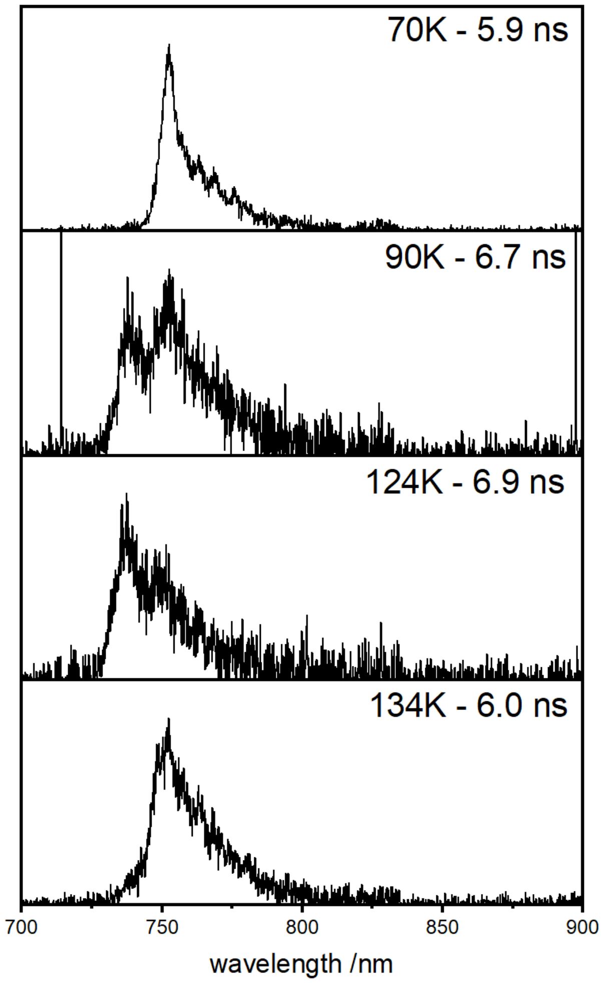

Figure S4. Spectral and correlated fluorescence lifetime fluctuations of a single DBT_h_{20} molecule. The temperatures and lifetimes are given in the upper right.

At 70 K the fluorescence spectrum of the molecule shown in Figure S4 was dominated by a single line in the region of the [0,0]-transition. At 90 K a second blue-shifted line came up due to spectral diffusion and got dominant at 124 K. In the spectrum at 134 K again only the red emission line was observed. The spectral changes were accompanied by significant alterations of the fluorescence lifetime which got longer with an increased contribution of the blue line. This behavior is a direct consequence of the EGL which predicts a decrease in the IC rate with a larger S_1-S_0 gap. Since similar behavior already had been observed at room temperature[2], these results show that the EGL can strongly affect the photophysics of individual molecules over a large temperature range.

References

[1] E. Clar, W. Willicks, *Chem. Ber.* 1955, **88**, 1205.
[2] C. Erker, Th. Basché, *J. Am. Chem. Soc*. 2022, **144**, 14053.
[3] K. Rurack, M. Spieles, *Anal. Chem.* 2011, **83**, 1232.
[4] ATTO740 Data Sheet, ATTO-TEC GmbH, **2021**.
[5] U. Resch-Genger, K. Rurack, *Pure Appl. Chem*. 2013, **85**, 2005.
